# Susceptibility of North American Ducks and Gulls to H5N1 Highly Pathogenic Avian Influenza Viruses

**DOI:** 10.3201/eid1211.060652

**Published:** 2006-11

**Authors:** Justin D. Brown, David E. Stallknecht, Joan R. Beck, David L. Suarez, David E. Swayne

**Affiliations:** * College of Veterinary Medicine, University of Georgia, Athens, Georgia, USA;; † Southeast Poultry Research Laboratory, Athens, Georgia, USA

**Keywords:** avian influenza virus, ducks, gulls, H5N1, highly pathogenic avian influenza, wild birds

## Abstract

Since 2002, H5N1 highly pathogenic avian influenza (HPAI) viruses have been associated with deaths in numerous wild avian species throughout Eurasia. We assessed the clinical response and extent and duration of viral shedding in 5 species of North American ducks and laughing gulls (*Larus atricilla*) after intranasal challenge with 2 Asian H5N1 HPAI viruses. Birds were challenged at ≈10 to 16 weeks of age, consistent with temporal peaks in virus prevalence and fall migration. All species were infected, but only wood ducks (*Aix sponsa*) and laughing gulls exhibited illness or died. Viral titers were higher in oropharyngeal swabs than in cloacal swabs. Duration of viral shedding (1–10 days) increased with severity of clinical disease. Both the hemagglutination-inhibition (HI) and agar gel precipitin (AGP) tests were able to detect postinoculation antibodies in surviving wood ducks and laughing gulls; the HI test was more sensitive than the AGP in the remaining 4 species

## Abstract 2

Species-related differences in clinical response and duration and extent of viral shedding exist between North American ducks and gulls infected with H5N1 HPAI viruses.

Free-living birds in the orders Anseriformes (ducks, geese, swans) and Charadriiformes (gulls, terns, shore birds) have traditionally been considered the natural reservoirs for avian influenza viruses (AIVs) (*1**,**2*). However, before 2005, no evidence showed that highly pathogenic avian influenza (HPAI) viruses were maintained in wild bird populations. Rather, HPAI viruses evolved independent of wildlife reservoirs when wild-type AIVs were introduced and adapted to domestic poultry populations (*3*). One exception occurred in 1961 when a high proportion of deaths in common terns (Sterna hirundo) in South Africa was attributed to an H5N3 HPAI virus without evidence of prior infection in domestic poultry (*4*). However, this tern epizootic was limited, and the virus did not become endemic in any wild bird population.

In 2002, a substantial number of deaths associated with H5N1 HPAI virus infection were reported in captive ducks, geese, and flamingos housed within 2 waterfowl parks in Hong Kong Special Administrative Region, People's Republic of China (*5*). Free-living gray herons (Ardea cinerea) and black-headed gulls (Larus ridbundus) also died during these outbreaks. Since 2002, sporadic deaths in wild birds, associated with H5N1 HPAI, have continued (*6*). Beginning in spring 2005, H5N1 HPAI outbreaks involving large numbers of wild birds were reported, and the subsequent spread of these viruses to Europe and Africa suggests that migratory birds may have been responsible for the long-range movement of these viruses. However, which wild avian species are important in H5N1 HPAI movement and whether these viruses will be established in free-living avian populations is unknown. The goal of this study was to determine the susceptibility of critical species of North American waterfowl to 2 H5N1 HPAI viruses and the potential impact of these species on the epidemiology of the viruses in North America.

## Material and Methods

### Animals

Five species of indigenous North American ducks were used in this study: mallard (Anas platyrhynchos), northern pintail (Anas acuta), blue-winged teal (Anas crecca), redhead (Aythya americana), and wood duck (Aix sponsa). Species were selected to represent the diverse habitat and behavior of ducks in North America and included important AIV reservoirs (mallard), long-distant migrants (northern pintail and blue-winged teal), diving ducks (redhead), and birds that breed in both northern and southern areas of the United States (wood duck). All ducks used in this study were captive-bred and acquired at 10 to 16 weeks of age (Howell's Exotic Waterfowl, Muldrow, OK, USA). This age is consistent with premigration staging in the late summer or early fall when AIV prevalence peaks in wild waterfowl (*7*). Both male and female ducks were included in each species and were approximately equally represented.

Wild-caught gulls used in this investigation were acquired through the Southeastern Cooperative Wildlife Disease Study, University of Georgia (UGA), under federal permit. Nestling laughing gulls (Larus atricilla) were hand-caught in McIntosh County, Georgia, by personnel from the Georgia Department of Natural Resources and maintained at the College of Veterinary Medicine, UGA. At 12 weeks of age the gulls were transported to biosafety level 3–agriculture (BSL-3-Ag) facilities at the Southeast Poultry Research Laboratory (SEPRL), Agricultural Research Service, United States Department of Agriculture (USDA).

All birds used in this study were cared for in accordance with the guidelines of the Institutional Animal Care and Use Committee, as outlined in the Guide for the Care and Use of Agricultural Animals in Agricultural Research and Teaching (*8*) and under an animal use protocol approved by the Institutional Animal Care and Use Committee at both SEPRL and UGA. All experiments were performed in the USDA-certified BSL-3-Ag facility at SEPRL (*9*).

### Viruses

Two viruses were used in this study: A/Whooper Swan/Mongolia/244/05 (H5N1) (Mongolia/05) and A/Duck Meat/Anyang/01 (H5N1) (Anyang/01). The Mongolia/05 isolate was obtained from a dead whooper swan (Cygnus cygnus) and was chosen because of its known lethality in wild waterfowl. The Anyang/01 isolate was chosen on the basis of results from previous experimental infections of Pekin white ducks (Anas platyrhyncos), which did not result in illness or death (*10*).

Individual stocks of both AIVs used in this study were produced by second passage in 9-day-old embryonated chicken eggs. Allantoic fluid from the inoculated eggs was diluted in brain-heart infusion (BHI) medium to yield a final titer of 10^6^ embryo 50% infectious doses (EID_50_) per 0.1 mL (single bird inoculum). A sham inoculum was prepared by diluting sterile allantoic fluid 1:30 in BHI.

### Experimental Design

Preinoculation serum was collected from each bird to confirm they were serologically naïve to influenza A viral antigens by agar gel precipitin test (AGP) and to H5 influenza by specific hemagglutination inhibition (HI) testing by using standard procedures (*11*). In addition, oropharyngeal and cloacal swabs were collected before inoculation to confirm an AIV-free status. The 5 species of ducks and laughing gulls were each separated into a control group and 2 virus-inoculated groups (Mongolia/05 and Anyang/01), each consisting of 3 birds. Ducks and gulls were inoculated intranasally with a 0.1-mL volume of the designated virus solution or sham-inoculum. All birds were monitored daily for illness or death. Due to the lack of illness exhibited by most ducks, experiments with these species were extended to 20 days postinoculation (DPI) to allow adequate time for seroconversion. Cloacal and oropharyngeal swabs were collected in BHI medium with antimicrobial drugs (100 μg/mL gentamicin, 100 units/mL penicillin, and 5 μg/mL amphotericin B) from all birds at 1, 2, 3, 4, 5, 7, 10, and 14 DPI. Oropharyngeal and cloacal swabs were also collected on 20 DPI from the 5 species of ducks. At 14 DPI (gulls) or 20 DPI (ducks), serum was collected from the surviving birds for serologic testing with HI and AGP, and the birds were humanely killed with intravenous sodium pentobarbital (100 mg/kg bodyweight). Serum was not collected from birds that died during the course of the study (that were not killed at the end of the study). Necropsies were performed on all birds, and routine tissues were collected for histopathologic and immunohistochemical evaluation. In addition, portions of heart, breast muscle, kidney, lung, and brain, and oropharyngeal and cloacal swabs were collected and stored in BHI medium with antimicrobial drugs for virus isolation.

### Histopathologic and Immunohistochemical Analysis

Tissues samples collected at necropsy were preserved in 10% neutral buffered formalin. After fixation, the tissues were routinely processed and embedded in paraffin. Sections were cut at a thickness of 5 μm and stained with hematoxylin and eosin. Duplicate sections were immunohistochemically stained by using a mouse-derived monoclonal antibody (P13C11) specific for type A influenza virus nucleoprotein antigen as the primary antibody (SEPRL, Athens, GA, USA). Procedures used to perform the immunohistochemical testing followed those previously described (*12*). Fast red was used as the substrate chromagen, and slides were counterstained with hematoxylin. Demonstration of viral antigen was based on chromagen deposition in the nucleus, with or without chromagen deposition in the cytoplasm.

### Virus Isolation

Oropharyngeal and cloacal swabs and tissue samples collected at necropsy were stored at -70°C until virus isolations and titrations were performed. Isolation of virus from swabs and tissues was performed by using embryonated chicken eggs (*11*). Positive samples were titrated by determining the EID_50_. The minimal detectable titer was 10^0.97^ EID_50_/mL from swabs and 10^1.97^ EID_50_/g from tissues.

### Serologic Assays

AGP and HI tests were performed on the pre- and postinoculation serum by using standard procedures (*11*). The HI tests were performed by using a 0.5% suspension of chicken erythrocytes in phosphate-buffered saline.

### Phylogenetic Analysis

In addition to the 2 H5N1 viruses used in this study, A/chicken/Hong Kong/220/97 (H5N1) (Hong Kong/97) was included in the phylogenetic analysis because it is the only other H5N1 HPAI virus evaluated in multiple avian species by experimental inoculation (*13*). Sequence comparisons of these 3 viruses were conducted with the MegAlign program by using the ClustalV alignment algorithm (DNASTAR, Madison, WI, USA), and phylogenetic relationships were estimated by the method of maximum parsimony (PAUP software, version 4.0b10; Sinauer Associates, Inc, Sunderland, MA, USA) by using a bootstrap resampling method with a heuristic search algorithm. Pairwise sequence comparisons were done within the MegAlign program.

## Results

### Morbidity and Mortality Data

**Figure 1:**
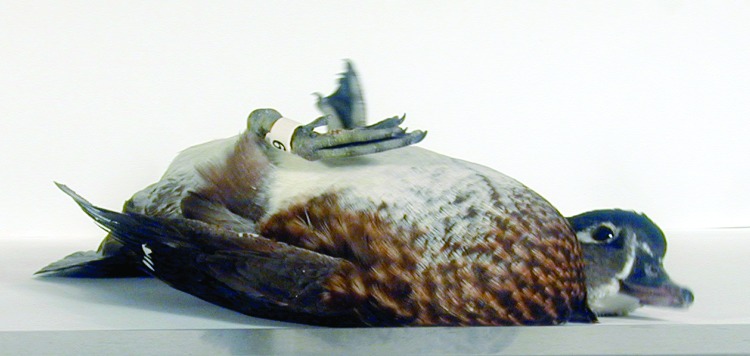
A female wood duck with severe neurologic clinical signs of disease after intranasal inoculation with an Asian strain of highly pathogenic avian influenza H5N1 virus.

Morbidity and mortality data are summarized in Table 1. Wood ducks were the only species of duck to exhibit illness or death after inoculation with either of the HPAI viruses. Severe clinical disease developed in 2 Mongolia/05-inoculated wood ducks, characterized by cloudy eyes, ruffled feathers, rhythmic dilation and constriction of the pupils, severe weakness, incoordination, tremors, and seizures (Figure 1). One of these ducks died at 7 DPI and the other was humanely killed at 8 DPI because of its moribund condition. Two Anyang/01-inoculated wood ducks became ill with clinical signs similar to those described for the Mongolia/05 virus group. One of these ducks died, and the other slowly recovered over 7 days until it exhibited no clinical symptoms. One wood duck in each viral group remained clinically normal for the entire trial. Clinical signs were not observed in the remaining duck species.

**Table 1:** Morbidity, mortality, and virus isolation data from 5 species of ducks and laughing gulls* intranasally inoculated with 2 different H5N1 HPAI viruses†

Virus/Host	No. sick/total (‡)	No. dead/total (§)	Virus isolation (oral swab)			Virus isolation (cloacal swab)		
			Prevalence, no. positive/total	Duration, days	AMT¶ (log_10_ EID_50_/mL)	Prevalence, no. positive/total	Duration, days	AMT (log_10_ EID_50_/mL)
Mongolia/05								
BWT	0/3	0/3	3/3	2	3.8	1/3	1	1.0
RD	0/3	0/3	3/3	1–4	2.8	2/3	1	1.2
WD	2/3 (5)	2/3 (7,8)	3/3	4–6	4.6	2/3	2,3	3.8
MD	0/3	0/3	3/3 (1)	1–3	3.1	1/3 (1)	1	1.0
NP	0/3	0/3	3/3	1–2	1.5	1/3	1	1.0
LG	3/3 (2–5)	2/3 (7,8)	3/3	7–8	4.2	3/3	4–7	2.6
Anyang/01								
BWT	0/3	0/3	2/3	1,2	2.0	0/3	–	–
RD	0/3	0/3	2/3	4	4.0	0/3	–	–
WD	2/3 (6)	1/3 (8)	3/3	7	5.0	2/3	4,5	2.8
MD	0/3	0/3	3/3	1–2	2.1	1/3	1	1.0
NP	0/3	0/3	2/3	1,4	1.1	0/3	–	–
LG	3/3 (3–5)	2/3 (9–10)	3/3	6–10	5.0	3/3	3–6	2.0

*Intranasally sham-inoculated control birds for each avian species lacked clinical, serologic, virologic, and pathologic evidence of avian influenza virus infection. †HPAI, highly pathogenic avian influenza; BWT, blue-winged teal; RD, redhead; WD, wood duck; MD, mallard; NP, northern pintail, LG, laughing gull; Mongolia/05, A/Whooper Swan/Mongolia/244/05; Anyang/01, A/Duck Meat/Anyang/01. ‡No. in parentheses indicates the first day postinoculation that clinical disease was apparent. §No. in parentheses indicates day of death. ¶Average maximum titer (AMT) is the average peak titer for birds that shed virus (log_10_ 50% embryo infective dose/mL).

All 3 Mongolia/05-inoculated laughing gulls exhibited severe clinical signs consisting of cloudy eyes, ruffled feathers, weakness, and incoordination, torticollis, or both. Two of these gulls died. The remaining gull clinically improved and stabilized over 6 days, but retained a head-tilt for the remainder of the trial. Severe clinical signs developed in all Anyang/01-inoculated gulls, similar to those seen in Mongolia/05-inoculated gulls. The disease progressed to death in 2 of these gulls. The remaining gull exhibited clinical signs for 8 days but gradually recovered until it showed no clinical symptoms.

### Pathologic Features

Viral-induced lesions were found only in the wood ducks and laughing gulls that exhibited clinical signs. Lesions were mild in birds that recovered but were severe and widespread in birds that died or were humanely killed due to severe illness. For each species, the severity and distribution of lesions were the same for both H5N1 viruses, with the following exception described below.

Gross lesions were not present in any of the recovered birds. Wood ducks that died had multiple petechial hemorrhages in the pancreas, whereas laughing gulls had more widely distributed petechial hemorrhages in the ventriculus, apex of the heart, cerebrum, and pancreas.

**Figure 2:**
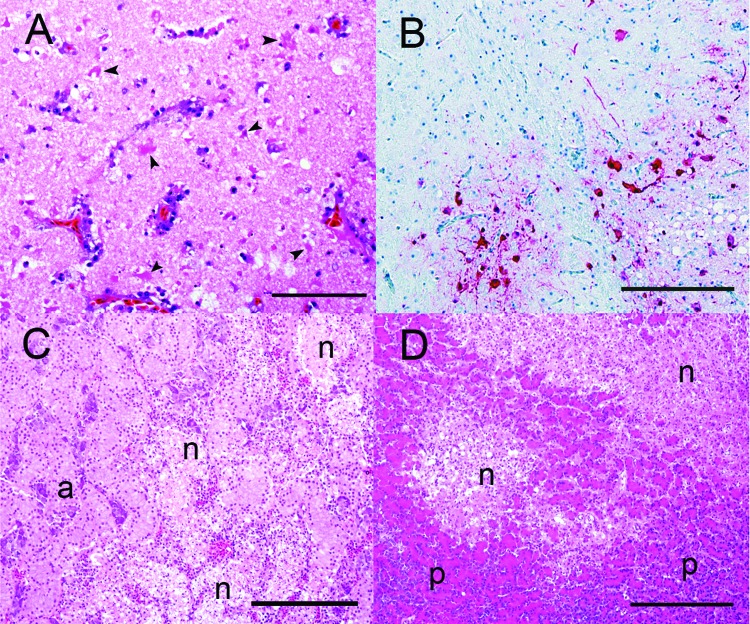
Photomicrographs of visceral organs from a wood duck that died after intranasal inoculation with a highly pathogenic avian influenza H5N1 virus. A) Brain with severe, multifocal to coalescing neuronal necrosis. Note the numerous necrotic neurons (arrowheads). Hematoxylin and eosin (HE) stain; bar =100 μm. B) Brain. Note the viral antigen (red) detected in the nucleus of several neurons. The unaffected brain tissue is blue. Immunohistochemical stain with hematoxylin counterstain; bar = 200 μm. C) Adrenal gland with necrotizing adrenalitis. Note the multiple foci of necrosis (n) surrounded by normal adrenal parenchyma (a). HE stain; bar = 200 μm. D) Pancreas with necrotizing pancreatitis. Note the 2 well-demarcated areas of necrosis (n) within the normal pancreatic tissue (p).

On histopathologic examination, wood ducks that died had severe, diffuse neuronal necrosis in the cerebrum (Figure 2A) and, less commonly, in the cerebellum. Other common lesions included necrotizing pancreatitis (Figure 2D) and adrenalitis (Figure 2C) and multifocal myocardial necrosis. Myocardial necrosis was only observed in wood ducks inoculated with Mongolia/05 and not with Anyang/01. Necrotizing pancreatitis and cerebral neuronal necrosis were the most common lesions in gulls that died during the study. Necrotizing adrenalitis was also observed in gulls that died but was less common and milder than the changes in the pancreas and cerebrum. Microscopic lesions in wood ducks and laughing gulls that recovered were less severe than in those that died. In both species, the most common lesions in recovered birds were lymphoplasmacytic perivascular encephalitis and heterophilic pancreatitis.

Wood ducks that died during the study had viral antigen in numerous organs, including the brain (Figure 2B), adrenal glands, testicles, kidneys, liver, small intestines, heart, skeletal muscles, pancreas, and air sacs. Viral antigen was most frequently found in cardiac myocytes, parasympathetic ganglia in the submucosal and muscular plexus of the small intestines, and numerous cell types in the brain, including glial cells, ependymal cells, endothelial cells, neurons, and gitter cells. Viral antigen was also detected in the pancreatic acinar cells and cortical and medullary cells of the adrenal gland, although less often than the aforementioned sites. Minimal amounts of viral antigen were detected in the kidney and testis in 1 and 2 wood ducks that died, respectively. The 1 wood duck that recovered had a scant amount of viral antigen in the cerebellar neurons. Laughing gulls that died during the study had viral antigen most frequently detected in the neurons, endothelial cells, glial cells, and ependymal cells in the brain, pancreatic acinar cells, and cortical and medullary cells of the adrenal glands. Laughing gulls that died also had minimal amounts of viral antigen present in other organs including the heart, lungs, air sacs, thymus, kidneys, small intestines, and eyes. Laughing gulls that recovered contained small amounts of viral antigen in the pancreatic acinar cells and cerebral and cerebellar neurons.

### Virus Isolation and Serologic Testing

The virus isolation results are summarized in Table 1 and Table 2. Viral titers were higher in oropharyngeal swabs than in cloacal swabs in all species and with both H5N1 viruses. Viral titers on cloacal swabs were low, except from birds that died of AIV infection. Oropharyngeal swabs from all species collected at 1 and 2 DPI were positive on virus isolation. Wood ducks and laughing gulls had higher viral titers on oropharyngeal and cloacal swabs and shed virus longer than any of the other species. Virus was isolated from numerous organs in the wood ducks and laughing gulls that died.

**Table 2:** Mean viral titer for tissues from wood ducks and laughing gulls* that died after inoculation with 2 different HPAI H5N1 viruses†

Host virus	Brain	Heart	Lung	Skeletal muscle	Kidney
WD-Anyang	3.7‡	3.5	5.1	ND§	2.9
WD-Mongolia	6.6	2.7	7.1	2.5	6.7
LG-Anyang	4.8	4.7	5.2	2.5	4.2
LG-Mongolia	6.3	2.5	3.3	4.2	2.5

*No virus was isolated from the internal organs of the other 4 avian species inoculated with H5N1 viruses and all of the sham-inoculated control birds. †HPAI, highly pathogenic avian influenza; WD, wood duck; LG, laughing gull; Anyang, A/Duck Meat/Anyang/01; Mongolia, A/Whooper Swan/Mongolia/244/05. ‡log_10_ mean 50% embryo infectious dose per gram (log_10_EID_50_/g). §Not detected.

Serologic testing results are summarized in Table 3. Both the AGP and HI tests detected postinoculation antibodies in all surviving wood ducks and laughing gulls. However, the effectiveness of these tests in the remaining duck species was variable and dependent on host species and inoculated virus. The HI test detected postinoculation antibodies in multiple avian species that had little serologic response or none as determined by the AGP test (Anyang/01-inoculated mallards, redheads, and northern pintails and Mongolia/05-inoculated mallards). Both serologic tests were least effective in northern pintails and mallards.

**Table 3:** Serology data from 5 duck species and laughing gulls inoculated with 2 different H5N1 HPAI viruses*

Virus/host	AGP serology		HI serology	
	Prechallenge, no. positive/total	Postchallenge, no. positive/total	Prechallenge, no. positive/total (GMT†)	Postchallenge, no. positive/total (GMT†)
Mongolia/05				
BWT	0/3	3/3	0/3	3/3 (13)
RD	0/3	3/3	0/3	3/3 (26)
WD	0/3	1/1	1/3 (8)	1/1 (128)
MD	0/3	0/3	0/3	1/3 (64)
NP	0/3	0/3	0/3	0/3
LG	0/3	1/1	1/3 (8)	1/1 (64)
Anyang/01				
BWT	0/3	0/3	0/3	3/3 (10)
RD	0/3	1/3	0/3	3/3 (20)
WD	0/3	2/2	0/3	2/2 (64)
MD	0/3	0/3	0/3	2/3 (16)
NP	0/3	0/3	0/3	2/3 (8)
LG	0/3	1/1	0/3	1/1 (32)

*HPAI, highly pathogenic avian influenza virus; AGP, agar gel precipitin; HI, hemagglutinationinhibition; BWT, blue-winged teal; RD, redhead; WD, wood duck; MD, mallard; NP, northern pintail; LG, laughing gull; Anyang/01, A/Duck Meat/Anyang/01; Mongolia/05, A/Whooper Swan/Mongolia/244/05. †GMT, geometric mean titer.

### Molecular Biology

In comparing the 3 viruses (Hong Kong/97, Anyang/01, and Mongolia/05) genetically, the hemagglutinin genes are all clearly in the Goose/Guandong/96 lineage. At the amino acid (aa) level they vary by 3.5%–4.8%. They all have the HA cleavage compatible with an HPAI phenotype. The cleavage site is the same for Hong Kong/97 and Anyang/01, but the Mongolia/05 virus has 2 aa changes at the cleavage site. Phylogenetically, Hong Kong/97 and the Anyang/01 are in or close to clade 3, and the Mongolia/05 strain is in clade 2 (*14*). The Mongolia/05 strain appears to be a representative isolate from the wild bird viruses that have been reported in Asia, Europe, and Africa.

Comparison of the other 7 gene segments demonstrates evidence of reassortment. The viruses from the 1997 outbreak in Hong Kong have a unique subtype-1 neuraminidase gene compared with any of the other H5N1 viruses. The Anyang/01 and Mongolia/05 N1 genes are from the same lineage, and both have an identical 20 aa stalk deletion. For the other 6 internal genes, the Anyang/01 and Mongolia/05 viruses in general were more closely related to each other than to the Hong Kong/97 virus. Except for the H5 gene, the Hong Kong/97 and viruses isolated in Hong Kong in the same year appear to be a unique constellation of genes that has not been seen again. Although the Anyang/01 and Mongolia/05 viruses were more closely related, the internal genes are most likely the result of reassortment with other influenza viruses and not the result of progressive sequence in a single lineage of viral genes.

## Discussion

Data from this study indicate that wood ducks and laughing gulls are highly susceptible to infection with H5N1 HPAI viruses as evidenced by widespread microscopic lesions, prolonged and highly concentrated viral shedding, and seroconversion. In addition, these species are likely to exhibit clinical disease or death associated with H5N1 virus infection. In a previous study, 2- to 3-week-old laughing gulls inoculated with A/chicken/Hong Kong/220/97 (H5N1) and A/tern/South Africa/61 (H5N3) did not exhibit illness or death (*15*). Viral replication in these birds was minimal and restricted to the respiratory tract. Since 2002, some emergent H5N1 viruses have exhibited unique characteristics, including lethality for waterfowl (*16*). Consistent with previous studies of ducks (*17*), the more recent isolates of H5N1 viruses used in our study caused a high proportion of illness and death in gulls, whereas the earlier H5N1 isolate, mentioned above, did not. To our knowledge, this is the first experimental inoculation of wood ducks with any HPAI viruses. Our results are consistent with field data that also indicate that wood ducks are highly susceptible to H5N1 HPAI viruses. In an investigation of H5N1 virus outbreaks in 2 waterfowl parks in Hong Kong, 18 of the 26 wood ducks on the lakes died (*5*). Of the wood ducks that died, 16 were positive for H5N1 virus by culture.

Traditionally, ducks asymptomatically shed high concentrations of wild-type AIVs in their feces (*18*). In this paradigm, ducks can transmit AIV over great distances as they migrate, and these viruses can remain infectious for prolonged periods of time in water (*18**,**19*). This fecal-oral mechanism is efficient at maintaining these viruses within duck populations and also transmitting AIVs from wild ducks to domestic poultry. Predominant oropharyngeal shedding has been consistently demonstrated with these H5N1 HPAI viruses (*20*), as it was in our study, and what impact this shedding pattern may have on environmental contamination, persistence in aquatic habitats, and transmission between birds (both wild and domestic) is unknown.

An efficient surveillance system is central to any preparedness program aimed to detect H5N1 in North America. Our data indicate that wood ducks and laughing gulls would be sensitive indicators of the presence of H5N1 circulating in wild birds. Wild avian species have previously been included in monitoring programs for other infectious diseases, for example, crow deaths for detection of West Nile virus (*21*). Other wild avian species in North America would also likely serve as sensitive indicators, but predicting which species is not possible without experimental inoculations or consistent morbidity and mortality data from outbreaks.

In relation to wood ducks and laughing gulls, the remaining 4 duck species were much less susceptible to H5N1 HPAI virus infection and were refractory to disease. Although these species may possibly contribute to viral transmission in wild avian populations, their role in the spread or maintenance of H5N1 HPAI virus is probably minimal. However, our experimental results are based on small sample sizes (n = 3) that are inadequate to fully evaluate potential individual bird variation in response to H5N1 challenge.

Illness, deaths, and viral shedding were less in our study than what has previously been reported for experimental inoculation of ducks with H5N1 HPAI virus. Possible explanations for the reduced pathogenicity include the age of birds used in the study and the variability between different H5N1 HPAI viruses. An age-dependent reduction in lethality was present between 2- and 4-week-old ducks inoculated intranasally with H5N1 HPAI virus (*22*). Similarly, previous experimental infections of mallards 2- to 6-weeks old with H5N1 resulted in a higher proportion of deaths than we observed with 10- to 16-week-old ducks (*17**,**20**,**23*). Experimental infections of very young ducks may overestimate the susceptibility of a species and the results may be incongruent with morbidity and mortality field data. The reduced pathogenicity and infectivity could also be characteristic for the specific H5N1 viruses used in this study.

One wood duck and 1 laughing gull reacted positively for preinoculation antibodies to AIV by the HI test. However, both of these birds were positive at the lowest detectable limit of this test, and these results may have been false-positive due to nonspecific hemagglutination. The wood duck did not become sick after inoculation with the Mongolia/05 isolate. The laughing gull did become ill after inoculation with the Anyang/01 virus, but completely recovered. If these serologic results are true positives, it is possible that the low antibody titers provided some immunologic resistance for these birds.

Serologic techniques commonly used in domestic poultry have limitations in ducks. The results of this study suggest variation between wild duck species in the ability of the AGP and HI tests to detect antibodies to type A influenza virus and H5 AIVs, respectively. Although the HI test was more sensitive than the AGP in detecting antibodies in our study, both serologic tests did not detect antibodies to AIV in some postinoculation serum samples from experimentally infected ducks. Furthermore, when duck erythrocytes were used in place of chicken erythrocytes for the HI test, antibodies were not detected in some of the duck samples (J. Brown, unpub.data). Surveillance systems that rely on these serologic techniques to detect H5N1 HPAI virus in ducks may substantially underestimate the prevalence of virus. In addition, false-positive results are possible if HI testing is used in H5N1 surveillance because positive results for H5 AIVs indicate previous infection with H5N1 HPAI virus or any other H5 wild-type AIV. Further information is necessary to evaluate the efficacy of these serologic assays in other wild avian species to allow correct interpretation of serologic field data.

The genetic sequence information indicated that all 3 evaluated H5N1 HPAI viruses were genetically distinct from each other, although the Anyang/01 and Mongolia/05 viruses were overall more closely related to each other than to Hong Kong/97. The only gene segment that all 3 viruses shared as part of a single viral lineage was the hemagglutinin gene. All 3 viruses had cleavage sites compatible with HPAI viruses, and experimental inoculation showed them to be extremely virulent in chickens (10,24, D. Swayne, unpub. data). Because of the large sequence differences, which genetic changes account for the virulence or host specificity differences cannot be identified. Reverse genetics has shown that single amino acid differences can greatly affect virulence, such as the change of glutamine to lysine at position 627 in the PB2 gene. This single difference can greatly increase the virulence of Hong Kong/97 viruses in mice (*25*).

The results of this study indicate that there is significant species-related variation in susceptibility, clinical disease, and antibody response to H5N1 virus infection in wild birds. Predicting this susceptibility beyond the species examined in this study is not possible. Wood ducks and laughing gulls were highly susceptible to H5N1 HPAI viruses with substantial illness and death. If H5N1 were introduced into North America, these species may serve as effective indicator species in a surveillance program.
