# Correction: Vol. 12, No. 11

**DOI:** 10.3201/eid1301.061301

**Published:** 2007-01

In "Susceptibility of North American Ducks and Gulls to H5N1 Highly Pathogenic Avian Influenza Viruses," by Justin D. Brown et al., an error occurred. In Table 1, morbidity, mortality, and virus isolation data for Mongolia/05 H5N1 HPAI virus from mallard ducks were omitted.

The corrected table appears in the updated article at http://www.cdc.gov/ncidod/EID/vol12no11/06-0652-T1.htm

We regret any confusion this error may have caused.

